# Decisões dos governos estaduais e os impactos na atenção à saúde de pessoas idosas na pandemia de COVID-19 no Nordeste brasileiro

**DOI:** 10.1590/0102-311XPT183023

**Published:** 2025-02-24

**Authors:** Vyna Maria Cruz Leite, Alberto Novaes Ramos, Anderson Fuentes Ferreira, Nicolas Gustavo Souza Costa, Carmem Emmanuely Leitão Araújo

**Affiliations:** 1 Faculdade de Medicina, Universidade Federal do Ceará, Fortaleza, Brasil.

**Keywords:** Idoso, COVID-19, Pandemia, Mortalidade, Políticas Públicas, Aged, COVID-19, Pandemic, Mortality, Public Policies, Anciano, COVID-19, Pandemia, Mortalidad, Políticas Públicas

## Abstract

Durante a pandemia de COVID-19, políticas de atenção à saúde para a população idosa ganharam relevância em todo o mundo. Com número de óbitos atingindo marcadamente essa população, torna-se importante analisar os impactos na atenção à sua saúde. O objetivo do artigo foi analisar a relação entre padrões de morbimortalidade relacionada à COVID-19 em pessoas com 60 anos ou mais de idade e medidas de prevenção e controle implementadas, a saber, distanciamento social e início da vacinação específica contra COVID-19, por estados da Região Nordeste do Brasil entre fevereiro de 2020 e setembro de 2022. Trata-se de estudo descritivo de série temporal fundamentado em dados dos estados da Região Nordeste. Os resultados apontam para elevado padrão de morbimortalidade da população idosa antes a pandemia de COVID-19 e crescimento acentuado após a emergência sanitária em todos os estados. Há certa homogeneidade entre as medidas de enfrentamento à pandemia adotadas pelos estados nordestinos. Houve incremento do número de internações com evolução para óbito em virtude da doença, mesmo após rigorosas medidas de contingência, distanciamento e início da vacinação. Concluiu-se que há uma relevante relação entre medidas sanitárias de distanciamento social e vacinação e redução de morbimortalidade da população idosa nos estados da Região Nordeste. Entretanto, as decisões dos governos estaduais da região não foram suficientes para mitigar o impacto da morbimortalidade entre as pessoas idosas em consequência da pandemia de COVID-19.

## Abstract (en)

During the COVID-19 pandemic, health care policies for older adults gained relevance worldwide. The number of deaths that markedly affected this population needs analyses of its impacts on health care. This study aimed to analyze the relation between COVID-19-related morbidity and mortality patterns in people aged 60 years and older and the implemented prevention and control measures, namely, social distancing and the beginning of specific vaccination against COVID-19 according to states in the Brazilian Northeast from February 2020 to September 2022. This descriptive time series study was based on data from the states in the Brazilian Northeast. Results point to a high pattern of morbidity and mortality in older adults before the COVID-19 pandemic, growing sharply after the onset of the pandemic in all evaluated states. The measures the states in the Brazilian Northeast adopted to combat the pandemic showed a certain homogeneity. The number of hospitalizations increase with the outcome death due to COVID-19, even after strict contingency and distancing measures and the beginning of vaccination. This study found a relevant relationship between sanitary measures of social distancing and vaccination and the reduction of morbidity and mortality in older adults living in the states in the Brazilian Northeast. However, the decisions of Northeastern states failed to mitigate the impact of morbidity and mortality in older adults to the COVID-19 pandemic.

## Abstract (es)

Durante la pandemia del COVID-19, las políticas de atención sanitaria para los adultos mayores cobraron relevancia a nivel mundial. Como las muertes afectaron considerablemente a esta población, es importante analizar los impactos en la atención sanitaria. El objetivo de este artículo fue analizar la relación entre los patrones de morbimortalidad relacionados con el COVID-19 en personas de 60 años o más y las medidas de prevención y control implementadas, a saber, el distanciamiento social y el inicio de la vacunación contra el COVID-19, por parte de los estados de la Región Nordeste de Brasil entre febrero de 2020 y septiembre de 2022. Este es un estudio descriptivo de series de tiempo, con base en datos de los estados de la Región Nordeste. Los resultados apuntan a una alta morbimortalidad de la población anciana antes de la pandemia del COVID-19 con un marcado crecimiento después de la emergencia sanitaria en todos los estados. Existe una cierta homogeneidad entre las medidas para combatir la pandemia adoptadas por los estados del Nordeste. Hubo un aumento de las hospitalizaciones con evolución a muerte por COVID-19, incluso después de las estrictas medidas de contingencia, distanciamiento y el inicio de la vacunación. Se concluyó que existe una relación relevante entre las medidas sanitarias de distanciamiento social y vacunación, y la reducción de la morbimortalidad de la población anciana en los estados de la Región Nordeste. Sin embargo, las decisiones de los gobiernos de los estados del Nordeste no fueron suficientes para mitigar el impacto de la morbimortalidad entre las personas mayores como resultado de la pandemia del COVID-19.

## Introdução

Impulsionadas pelos desafios impostos pela pandemia da doença relacionada ao novo coronavírus de 2019 (COVID-19), as políticas de atenção à saúde e de proteção social voltadas para a população idosa (no Brasil, aquelas com 60 anos ou mais de idade) passaram a ter maior relevância globalmente. Considerando a elevada letalidade registrada nessa população, ações e iniciativas governamentais precisaram ser definidas de forma custo-efetiva e oportuna de políticas públicas, não somente na atenção à saúde, mas no combate a violências, na proteção social e na proteção à vida em diferentes contextos e realidades ^1^ ^,^ ^2^ ^,^ ^3^ ^,^ ^4^. O contexto brasileiro não foi diferente, uma vez que os óbitos associados à COVID-19 atingiram de modo desigual mais pessoas desse grupo, demandando uma análise mais cuidadosa acerca dos impactos na atenção à sua saúde. Para tanto, políticas públicas de proteção à vida em pessoas idosas demandaram maior eficiência nas diferentes realidades do país ^3^ ^,^ ^4^.

Para além da elevada transmissibilidade, a pandemia de COVID-19 trouxe outros aspectos críticos à discussão das políticas sociais no Brasil, tais como a preocupação frente à transição demográfica e seu tensionamento com a produtividade, o empobrecimento e o aumento das desigualdades sociais ^5^ ^,^ ^6^ ^,^ ^7^. A pandemia atingiu sobremaneira a população idosa, não apenas com a detecção de casos sintomáticos, mas também com a ocorrência de casos com maior letalidade, demasiados com a maior idade das pessoas acometidas ^8^. Marcadamente atingida pela pandemia de COVID-19, a vulnerabilidade de pessoas idosas se ampliou, com impactos diferenciais na qualidade e na expectativa de vida dessa população ^1^.

A idade tem sido apontada como um dos principais fatores que influenciam o status e a progressão da infecção por SARS-CoV-2 ^8^. O processo inflamatório e a imunossenescência reduzem a capacidade de resistência e representam fatores que contribuem para vulnerabilidade ampliada a doenças infecciosas, com impacto negativo frente a prognósticos desfavoráveis, em particular na vigência de condições crônicas ^5^ ^,^ ^9^. A presença de comorbidades pode influenciar a dinâmica do receptor da enzima conversora da angiotensina 2 (ACE2) e de diferentes citocinas, possibilitando não apenas maior transmissão e infecção, como também maior probabilidade de progressão da doença, particularmente diante da eventual fragilidade do estado mental de pessoas idosas ^8^.

É fato que o número de óbitos de COVID-19 teve rápido crescimento ao longo do tempo, particularmente na população mais idosa em contextos de maior vulnerabilidade social ^10^. Dados do *Painel Coronavírus* do Ministério da Saúde ^11^ até julho de 2023 indicaram o registro de mais de 705 mil óbitos de COVID-19, quase 70% em pessoas com idade acima de 60 anos. Outros fatores, como ser do sexo masculino e ter idade ainda mais avançada, estiveram associados à mortalidade entre pessoas idosas hospitalizadas acometidas de COVID-19 ^12^.

As intervenções para mitigar os impactos da pandemia de COVID-19 variaram substancialmente entre Unidades da Federação (UF) e municípios brasileiros, em relação tanto ao perfil de medidas adotadas quanto ao momento em que foram instituídas ^7^ ^,^ ^13^. A epidemia apresentou impactos de forma acentuada nos estados das regiões mais vulneráveis socialmente do país, particularmente Norte e Nordeste, com a última representando 27% da população brasileira e aproximadamente 20% de todos os casos de COVID-19 e dos óbitos em consequência da doença ^7^ ^,^ ^11^. As iniciativas de enfrentamento da pandemia a serem adotadas por governos estaduais para redução da morbimortalidade deveriam ter sido potencializadas em caráter prioritário nessas regiões onde a COVID-19 teve elevado impacto ^7^ ^,^ ^14^ ^,^ ^15^.

O presente artigo tem como objetivo analisar a relação entre padrões de morbimortalidade relacionada à COVID-19 em pessoas com 60 anos ou mais de idade e medidas de prevenção e controle implementadas, a saber, distanciamento social e início da vacinação específica contra COVID-19, por estados da Região Nordeste durante o período pandêmico.

## Métodos

### Delineamento e local do estudo

Trata-se de estudo de série temporal descritivo fundamentado em dados acerca da COVID-19 em estados da Região Nordeste. Foram analisados dados gerais de ocorrência de casos e óbitos na população com 60 anos ou mais de idade, além de internações hospitalares (incluindo evolução para óbito), tanto em caráter ambulatorial quanto de terapia intensiva, no período de 2018-2022. Foram considerados os dois anos anteriores e os dois posteriores ao primeiro ano da pandemia.

Os dados epidemiológicos em cada um dos estados da Região Nordeste foram confrontados com o registro documental da implementação de medidas de enfrentamento à COVID-19, particularmente o tempo entre o primeiro caso no Brasil e o primeiro decreto estadual de medidas distanciamento, bem como o tempo entre o decreto de distanciamento estadual e o início da vacinação.

Composta por nove estados (Alagoas, Bahia, Ceará, Maranhão, Paraíba, Pernambuco, Piauí, Rio Grande do Norte e Sergipe) e 1.793 municípios, a Região Nordeste possui, segundo dados do *Censo Demográfico* de 2022, 54,6 milhões de habitantes, representando 27% da população do país e sendo a segunda mais populosa no Brasil ^16^. Ainda segundo o Censo, 15,8% da população do país tem mais de 60 anos, ou seja, é idosa. De fato, a população de pessoas idosas residentes no Brasil era de mais de 32 milhões de pessoas, representando um acréscimo de 56% em relação à de 2010. Na Região Nordeste, 14,5% de sua população era idosa ^16^ ^,^ ^17^.

### Fonte e análise dos dados

Foram coletados dados demográficos de cada estado da Região Nordeste via *site* do Instituto Brasileiro de Geografia e Estatística (IBGE) ^16^ ^,^ ^17^, em termos de número e proporção de pessoas com 60 anos ou mais de idade em relação à população geral. Os dados de morbimortalidade de COVID-19, incluindo número de casos, óbitos, internações hospitalares e mortes durante internação hospitalar, foram coletados do *Painel Coronavírus* ^11^.

Dados da vacinação específica foram provenientes dos planos operacionais e registros oficiais de cada Secretaria Estadual de Saúde e consideradas como a data de início, a primeira dose aplicada em grupos prioritários.

As informações acerca das medidas de vigilância, prevenção e controle da COVID-19, especificamente voltadas para população idosa, foram obtidas a partir de Decretos Estaduais, sendo considerados os documentos de início de cada medida específica. Assim, foram considerados os Decretos: *nº 69.541* ^18^, de 19 de março de 2020, de Alagoas; *nº 19.529* ^19^, de 16 de março de 2020, da Bahia; *nº 33.510* ^20^, de 16 de março de 2020, do Ceará; *nº 35.660* ^21^, de 16 de março de 2020, do Maranhão; *nº 40.128* ^22^, de 17 de março de 2020, da Paraíba; *nº 48.809* ^23^, de 14 de março de 2020, de Pernambuco; *nº 18.884* ^24^, de 16 de março de 2020, do Piauí; *nº 29.524* ^25^, de 17 de março de 2020, do Rio Grande do Norte; e *nº 40.560* ^26^, de 16 de março de 2020, de Sergipe.

Os primeiros decretos de distanciamento social e do início da vacinação que incluíram a população idosa nos estados, à luz dos padrões de morbimortalidade associados à população idosa, antes e durante a pandemia de COVID-19, incluindo a letalidade associada à COVID-19, foram sistematizados e comparados entre as nove UF da Região Nordeste. Adicionalmente, buscou-se verificar variações entre os estados com vistas a reconhecer as medidas implementadas e as diferenças de implementação entre as UF no concernente à priorização dos grupos para início da vacinação ^27^. 

Os dados coletados foram consolidados, analisados descritivamente por meio de números absolutos e relativos, e apresentados na forma de tabela e gráficos utilizando o software Prism (https://www.graphpad.com/) Calculou-se ainda, por meio desse software, a taxa de mortalidade específica por COVID-19 a partir do número de óbitos ocorridos na população idosa em relação ao total dessa população residente, para fins comparativos entre os diferentes estados no Nordeste.

## Aspectos éticos

Os resultados deste estudo fazem parte de pesquisa aprovada pelo Comitê de Ética em Pesquisa envolvendo Seres Humanos da Universidade Federal do Ceará, em 18 de junho de 2021 (parecer nº 4.790.275).

## Resultados

Após o primeiro caso de COVID-19 ser confirmado no Brasil, em 26 de fevereiro de 2020, em estado localizado na Região Sudeste do país, registrou-se mais de duas semanas para os estados nordestinos apresentarem decisões mais contundentes sobre o enfrentamento da pandemia, de acordo com as orientações da Organização Mundial da Saúde (OMS) ^28^.

O Estado de Pernambuco foi o primeiro a publicar decreto com medidas de controle. Embora tenha adotado essa decisão 17 dias após o primeiro caso nacional, esse estado demorou apenas dois dias para o registro da notificação do caso autóctone. Alagoas, por sua vez, o último a publicar seu primeiro decreto de distanciamento social, demorou 23 dias a partir do primeiro caso brasileiro para tomada de decisão que permitiria o controle da transmissão comunitária, 12 dias depois do caso notificado no estado (Tabela 1).

**Tabela 1:** Morbimortalidade de COVID-19 e medidas de prevenção e controle adotadas por estados da Região Nordeste do Brasil, entre março de 2020 e agosto de 2022.

	Alagoas	Bahia	Ceará	Maranhão	Paraíba	Pernambuco	Piauí	Rio Grande do Norte	Sergipe
Total de casos (n)	236.963	1.225.258	935.273	351.917	436.528	620.723	317.817	365.874	277.611
Total de casos de pessoas com 60 anos ou mais de idade (n)	32.390	87.283	147.340	62.234	63.567	100.412	47.609	57.754	35.117
Proporção de casos em pessoas com 60 anos ou mais de idade (%)	13,66	7,12	15,75	17,68	14,56	16,17	14,98	15,78	17,64
Total geral de óbitos confirmados (n)	6.121	26.617	24.115	10.084	9.230	19.740	6.966	7.292	6.002
Óbitos de pessoas com 60 anos ou mais de idade por COVID-19 (n)	4.003	18.100	17.534	7.063	6.077	13.728	5.063	4.741	3.933
Proporção de óbitos por COVID-19 em pessoas com 60 anos ou mais de idade (%)	65,39	68,00	72,60	70,04	65,83	69,54	72,68	65,01	65,52
Primeiro caso de COVID-19	08/Mar/2020	06/Mar/2020	15/Mar/2020	20/Mar/2020	18/Mar/2020	12/Mar/2020	19/Mar/2020	19/Mar/2020	14/Mar/2020
Primeiro óbito por COVID-19	31/Mar/2020	29/Mar/2020	24/Mar/2020	29/Mar/2020	31/Mar/2020	25/Mar/2020	27/Mar/2020	28/Mar/2020	02/Abr/2020
Primeiro decreto estadual de medidas de distanciamento	*nº 69.541* ^18^ (19/Mar/2020)	*nº 19.529* ^19^ (16/Mar/2020)	*nº 33.510* ^20^ (16/Mar/2020)	*nº 35.660* ^21^ (16/Mar/2020)	*nº 40.128* ^22^ (17/Mar/2020)	*nº 48.809* ^23^ (14/Mar/2020)	*nº 18.884* ^24^ (16/Mar/2020)	*nº 29.524* ^25^ (17/Mar/2020)	*nº 40.560* ^26^ (16/Mar/2020)
Número de dias entre primeiro caso no Brasil e decreto de distanciamento estadual	23	20	19	19	22	17	19	17	20
Início da vacinação da população geral	17/Jan/2021	19/Jan/2021	18/Jan/2021	18/Jan/2021	19/Jan/2021	18/Jan/2021	18/Jan/2021	19/Jan/2021	19/Jan/2021

Fonte: elaboração própria.

Comparativamente, a proporção de pessoas infectadas por SARS-CoV-2 foi maior em pessoas com menos de 60 anos de idade em todos os estados nordestinos, a despeito do maior número de mortes na população idosa. A Bahia, apesar de ser o estado mais populoso, apresentou menor proporção de casos em pessoas com mais de 60 anos. Ceará e Pernambuco, segundo e terceiro estados em população, respectivamente, tiveram o maior número de pessoas idosas com diagnóstico de COVID-19.

Quando analisados os estados nordestinos com menor população (Sergipe, Piauí, Rio Grande do Norte e Alagoas), verificou-se que o Estado de Alagoas teve a menor proporção de casos em pessoas com mais de 60 anos em relação aos demais estados, apesar da maior proporção de óbitos nesta população (65,4%), superior a Sergipe (65,5%) e Rio Grande do Norte (65%). O Piauí foi o estado que apresentou o pior resultado nesse indicador na região (72,7%). Alagoas, último estado da região a adotar medidas oficiais para conter a pandemia com aumento crescente de casos confirmados da COVID-19, publicou seu primeiro decreto de isolamento social no dia 19 de março de 2020, 12 dias após confirmado o primeiro caso em seu próprio território. No dia 31 de março seria registrada a primeira morte de COVID-19 em Alagoas.

Quando considerados os óbitos em consequência da COVID-19 em pessoas acima de 60 anos, os estados do Piauí e Ceará apresentaram as maiores proporções, com 72,7% e 72,6%, respectivamente. No entanto, nenhum estado da região registrou proporções inferiores a 65% de óbitos pela COVID-19 nesse demográfico.

De forma semelhante, comportou-se o Estado da Paraíba, que decretaria a primeira medida para conter o aumento da pandemia 22 dias após confirmado o primeiro caso no país, assim como o Estado da Bahia, ao tornar pública a primeira medida de isolamento e distanciamento, 11 dias após a confirmação do primeiro caso em seu território. Doze dias após tal medida, a primeira morte seria confirmada pela COVID-19 na Bahia.

A Tabela 1 também traz informações acerca do início da vacinação nos estados da região, deflagrada no mês de janeiro de 2021. Verificou-se que os estados de Alagoas, Paraíba, Pernambuco, Piauí, Rio Grande do Norte e Sergipe iniciaram a vacinação pelo menos 15 dias após Bahia, Ceará e Maranhão, uma diferença significativa entre dois grupos de estados. Com base nos primeiros decretos estaduais da vacinação, não foram reconhecidas diferenças relevantes com relação aos grupos prioritários. Houve indicação técnica integrando profissionais de saúde e pessoas idosas institucionalizadas como prioritárias.

**Figura 1:**
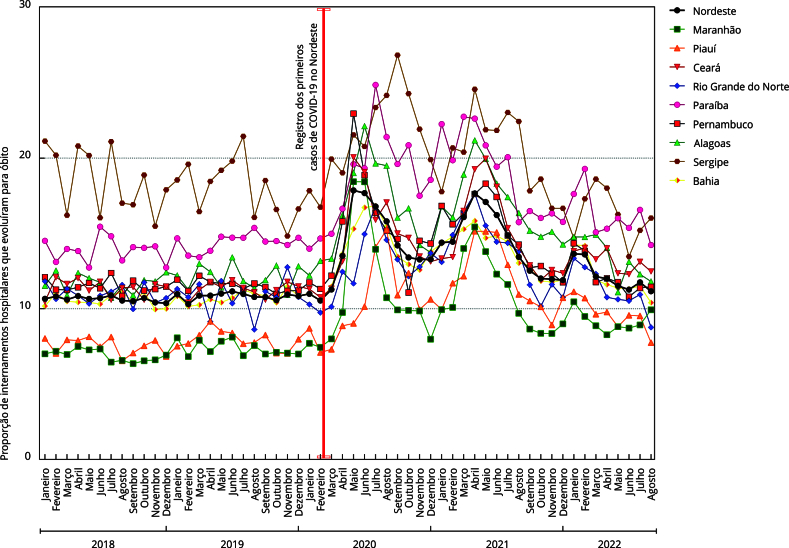
Proporção de internações hospitalares com evolução para óbito entre pessoas idosas, nos estados da Região Nordeste do Brasil, entre janeiro de 2018 e agosto de 2022.

Na Figura 1, apresenta-se a proporção de internações hospitalares (ambulatoriais e em UTI, por todas as causas) com evolução para óbito entre pessoas com 60 anos de idade ou mais, entre janeiro 2018 e setembro de 2022 na Região Nordeste. Em virtude da COVID-19, todos os estados da região apresentaram um importante incremento do número de internações com evolução para óbito em março 2020. O Estado de Sergipe já apresentava número maior de internações com evolução para óbitos desde 2018 se comparando com os demais, e manteve esse comportamento, sendo o estado com pior resultado, seguido por Paraíba e Pernambuco.

O Estado do Maranhão apresentou a menor proporção de internações hospitalares com evolução para óbitos entre pessoas idosas antes da pandemia, mas foi o sexto estado com pior evolução desse indicador durante o ano de 2020. Todos os estados apresentaram tendência a redução das internações hospitalares com evolução para óbito em pessoas idosas a partir de janeiro de 2021, a despeito de novo aumento de óbitos em meados de maio de 2021, após rigorosas medidas de contingência, distanciamento e uso obrigatório de equipamentos de proteção individual, bem como após o início da vacinação no país. Pernambuco e Paraíba, no entanto, mantiveram em patamares elevados as internações hospitalares com desfecho para óbito ainda em 2021.

**Figura 2:**
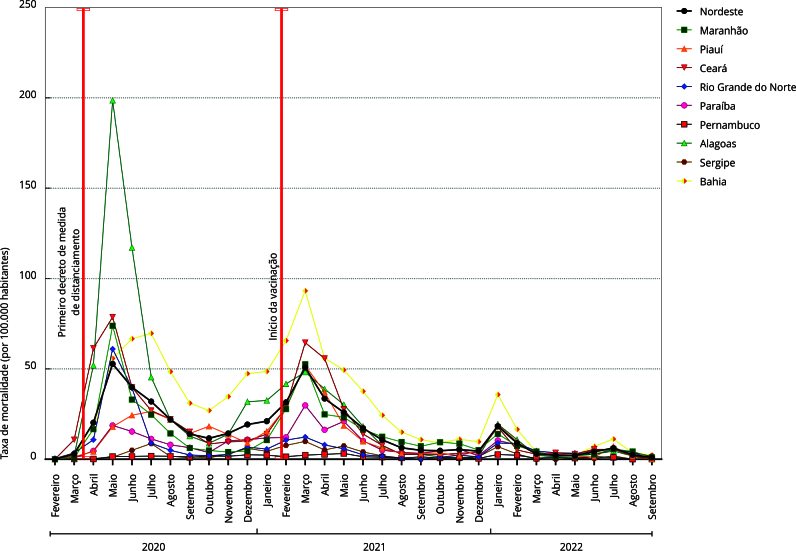
Taxa de mortalidade específica por COVID-19 (por 100 mil habitantes) em pessoas idosas nos estados da Região Nordeste do Brasil, entre fevereiro de 2020 e setembro de 2022.

A taxa de mortalidade por 100 mil habitantes na população idosa apresentou padrão epidemiológico semelhante entre os nove estados da região, com aumento rápido e expressivo entre os meses de março e abril de 2020 e pico no mês de junho de 2020. Padrão semelhante foi verificado entre os estados no final de 2021 e início de 2022, com tendência de redução da mortalidade, mas com platô de estabilidade ainda em níveis elevados. Entre os meses de junho e julho de 2020, todos os estados iniciaram tendência de redução das taxas de mortalidade, ou seja, cinco meses após as primeiras decisões estaduais de enfrentamento a pandemia, a divulgação dos planos de contingência, os decretos de estado de emergência e a adoção das primeiras medidas de distanciamento social. A Figura 2 nos traz a perspectiva que considera as taxas de mortalidade de COVID-19 na população idosa, que foram analisadas ao se observar duas imprescindíveis medidas para controle da pandemia de COVID-19, as primeiras publicações em decretos sobre o distanciamento social em cada estado e o início da vacinação na população prioritária nos estados nordestinos.

Nos seis primeiros meses de 2020, os estados do Maranhão, Ceará, Rio Grande do Norte, Alagoas e Bahia tiveram picos da taxa de mortalidade de COVID-19 em pessoas idosas, com quase 80 óbitos/100 mil habitantes, estando o estado de Alagoas com a pior situação com 200 óbitos/100 mil habitantes. Após as primeiras medidas de distanciamento social, os estados apresentaram redução consistente da mortalidade, particularmente entre os meses de maio de junho de 2020.

As medidas de distanciamento social precederam em dois meses a redução da taxa de mortalidade da população idosa na maioria dos estados (Maranhão, Ceará, Rio Grande do Norte, Paraíba e Alagoas). Entretanto, em outros se verificou retardo de quatro meses para registro decréscimo da mortalidade (Piauí, Bahia e Sergipe). Pernambuco não apresentou tendência significativa de declínio da taxa de mortalidade, visto que a redução em cinco meses depois da primeira medida de distanciamento não se manteve nos meses subsequentes. Entretanto, todos os estados apresentaram novo incremento da taxa de mortalidade nos primeiros meses de 2021, particularmente em fevereiro, logo após o momento em que a vacinação é iniciada em todo país. Como consequência, após entre um a dois meses, verificou-se significativa redução da mortalidade de pessoas idosas em todos os estados, exceto em Pernambuco, onde a taxa persistiu elevada até maio de 2021, verificando-se para decréscimo da mortalidade no local somente após três meses.

Durante o ano de 2022, período em que a população idosa já havia sido vacinada com pelo menos três doses com as vacinas disponíveis na rede pública do Sistema Único de Saúde (SUS), todos os estados apresentaram tendência significativa de redução da mortalidade de pessoas com mais de 60 anos por COVID-19.

## Discussão

O estudo traz evidências adicionais acerca do padrão diferencial de morbimortalidade relacionada à COVID-19 em pessoas idosas frente ao momento de adoção de medidas de prevenção e controle em estados da Região Nordeste durante o período pandêmico.

No Brasil, apesar de melhorias nas condições socioeconômicas da população com redução de desigualdades regionais, históricas iniquidades territoriais em saúde persistem ^29^ ^,^ ^30^. Com elevada densidade urbana que coexiste com a expansão de problemas antes limitados às grandes capitais ^31^, a crise sanitária nos estados nordestinos refletiu condicionantes de alta vulnerabilidade da região à COVID-19 ^32^. Há ainda fragilidades nas redes assistenciais ^33^, expressando dificuldades para atender as necessidades relacionadas ao envelhecimento da população ^34^. Ademais, o Nordeste concentra em números absolutos maior prevalência de pessoas idosas com multimorbidades de risco para COVID-19 grave ^12^. Essa moldura estrutural é relevante para entender a elevada carga de morbimortalidade de COVID-19 na população idosa no Nordeste brasileiro.

Diante de um problema de acentuada envergadura mundial, respostas rápidas para controle da disseminação da COVID-19 e para garantia de assistência adequada à saúde eram necessárias. Assim, inúmeras medidas se tornaram centrais, como a ampliação do número de leitos, a compra e disponibilização de insumos e de equipamentos de proteção individual, o estabelecimento de medidas de distanciamento social, a garantia de acesso à testagem e aos medicamentos necessários ao tratamento, e a garantia da vacinação ^35^ ^,^ ^36^ ^,^ ^37^.

Antes da pandemia de COVID-19, conforme aqui apresentado, a proporção de internações hospitalares com evolução para óbito entre pessoas com mais de 60 anos já era preocupante nos estados nordestinos. Apesar dos poucos estudos, as disparidades entre as necessidades de saúde da população idosa e a oferta de serviços para atendê-las eram notadas, de modo a sinalizar possíveis iniquidades no acesso antes do período pandêmico ^38^. É importante considerar, portanto, que diferenças nos padrões epidemiológicos podem sugerir que as proporções elevadas de óbitos de pessoas idosas antes e durante a COVID-19 estão relacionadas à ação pública.

Neste estudo, Sergipe e Paraíba registraram piores resultados na proporção de internações com evolução para óbito de pessoas idosas antes e depois da pandemia. Em contrapartida, outros estados, embora com melhor parâmetro de morbimortalidade de pessoas idosas nos anos de 2018 e 2019, ampliaram sua proporção de óbitos na COVID-19. Essa descontinuidade no padrão de morbimortalidade reforça que, para além das intervenções de controle em ocasião da crise sanitária, recursos humanos, físicos, materiais e mecanismos de atenção pré-existentes são fatores necessários para explicar uma resposta eficaz no contexto pandêmico ^39^. Entretanto, as estratégias de controle da transmissão adotadas pelos governos também podem gerar mudanças no desempenho ^40^ ^,^ ^41^ ^,^ ^42^.

Os achados deste estudo apontam ainda para a semelhança nas iniciativas dos estados nordestinos em relação ao enfrentamento da COVID-19. Os planos de contingência e as recomendações subsequentes, ainda que com singularidades, apresentaram conteúdos semelhantes, tanto na orientação do distanciamento social, quanto aos critérios para a vacinação. A literatura tem caracterizado a atuação dos estados nordestinos como rigorosa, à exceção da experiência do estado da Bahia, tida como branda ^43^. A homogeneidade das normativas na região tem sido atribuída a colaborações interestaduais em dispositivos colegiados, como o Consórcio Nordeste, assim como às semelhanças sociodemográficas e às aproximações político-partidárias ^7^ ^,^ ^43^ ^,^ ^44^ ^,^ ^45^.

Os decretos estaduais relacionados à pandemia de COVID-19 foram, em grande medida, formulados com base em aspectos técnico-administrativos ^46^, indo de encontro às orientações do Governo Federal. O uso de evidências científicas esteve presente no gerenciamento da pandemia dos estados nordestinos na formulação de planos de distanciamento social e de protocolos para setores econômicos específicos. Nessa direção, destaca-se ainda a capacidade de influência dos comitês estaduais de enfrentamento da COVID-19, em sua maioria constituídos de forma multidisciplinar ^44^.

Contudo, os achados desta pesquisa indicam um relativo atraso na formulação de respostas subnacionais à pandemia da COVID-19 que incidiu na morbimortalidade da população idosa. Em contexto de elevada gravidade e transmissibilidade da nova doença, mesmo antes da divulgação dos primeiros casos, três variantes de SARS-CoV-2 estavam distribuídas em todo país, das quais duas tiveram entrada pelo Estado do Ceará ^47^. Com a constatação do primeiro caso em território nacional com rápida difusão do vírus nos estados brasileiros ^48^, observam-se diferenças relevantes no tempo de estabelecimento de medidas de controle da doença pelos estados do Nordeste, considerando-se como ponto de referência os primeiros decretos estaduais.

Importante considerar que a quantidade de testes realizados pelos estados nordestinos foi considerada insuficiente para dimensionamento mais preciso da pandemia ^49^. Ademais, mudanças nos sistemas de notificação, limitações na disponibilidade de informações sobre a assistência privada e desigualdades no acesso aos testes no país incidiram na capacidade pública de avaliação e resposta em tempo real da transmissão do vírus ^50^. Essas fragilidades na vigilância epidemiológica podem ter contribuído com a demora na resposta estadual e, consequentemente, com a morbimortalidade pela COVID-19.

Problemas relacionados à oferta de serviços de saúde, cuidados e proteção à vida da população idosa ^51^, por certo, se intensificaram no período. Nos primeiros meses da pandemia, a ausência de vacinas e medicamentos antivirais específicos frente à elevada transmissibilidade ressaltou as medidas de distanciamento social e vigilância de casos como as principais intervenções eficazes para controle ^52^.

Em relação à implementação de medidas de distanciamento social, é relevante notar os inúmeros desafios socioeconômicos para a sustentabilidade das restrições de mobilidade, ou seja, de transmissibilidade dos vírus da síndrome respiratória aguda grave ^53^. A informalidade se soma à fragilidade dos vínculos trabalhistas, com implicações correlatas aos desafios das políticas de proteção social em contexto de emergência ^54^.

Nesse contexto, vale destacar a fragilidade laboral da população idosa brasileira, os quais em alta proporção se mantiveram trabalhando durante a pandemia, muitos em atividades informais com perdas significativas de renda, sendo maior entre pessoas já empobrecidas ^55^. Nesse sentido, pessoas idosas em áreas socialmente vulneráveis, em Serviço de Acolhimento Institucional para as Pessoas Idosas (ILPI) e em Situação de Rua apresentaram maior mortalidade ^56^ ^,^ ^57^. Ademais, pessoas idosas com multimorbidades, embora tenham apresentado a tendência de maior adesão ao distanciamento social, possivelmente enfrentaram a agudização de problemas crônicos ^58^.

Além das medidas não farmacológicas, a vacinação representou um importante avanço, decisivo para controle da pandemia, demonstrado aqui pela consequente redução de mortalidade na população idosa. Apesar de diferentes estudos demonstrarem reduzida hesitação vacinal nessa população ^59^, o uso das mídias sociais esteve associado a menores índices de intenção a se vacinar, considerando discursos negacionistas de descrédito da eficácia ^60^. Nos meses de agosto e setembro de 2021, muitas cidades brasileiras já tinham aplicado ao menos a primeira dose em praticamente 100% de todas as pessoas com mais de 18 anos ^61^ ^,^ ^62^. Como consequência, a proporção de pessoas acima de 60 anos que foram hospitalizados e que evoluíram para óbito apresentou redução durante o mesmo período ^40^. No entanto, o presente estudo evidencia que Pernambuco não apresentou tendência consistente de redução da mortalidade após o distanciamento social, além de demora para declínio desse indicador após o início da imunização. Isso sugere que especificidades locais e outros fatores intervenientes podem contribuir com padrões distintos de controle.

É preciso considerar ainda que o SUS possui mecanismos de compartilhamento de decisões e de definição de responsabilidades, que reforçam o papel da União como indutora de políticas nacionais de saúde em âmbito local ^6^ ^,^ ^35^. Entretanto, na crise sanitária, o Governo Federal apresentou limitada coordenação da política de saúde, complicada frente a confrontos políticos com as unidades subnacionais, o que exigiu a equalização pelo Supremo Tribunal Federal de reconhecer a autonomia dos governos estaduais na definição de medidas de enfrentamento de COVID-19 ^35^, com impactos junto as pessoas idosas na pandemia ^63^.

Os dados analisados não permitem uma discussão mais detalhada das possíveis causas da mudança na evolução de casos e óbitos na população idosa, o que se configura em uma das limitações da pesquisa, já que há outras possíveis causas da mudança na evolução dos óbitos nesta população, além dos decretos publicados por governos estaduais.

Em uma sociedade em que o envelhecimento populacional se processa aceleradamente, torna-se uma questão de saúde pública repensar condicionantes estruturais e contextuais para o adoecimento das pessoas mais velhas, especialmente em uma emergência sanitária que as põe em maior risco ^64^. Diferentes dimensões de vulnerabilidade se interconectam para a determinação dos efeitos de morbimortalidade na população idosa, de modo que as condições sociais e programáticas se associam na variação de fragilidades de saúde ^65^. Todavia, pessoas idosas foram desproporcionalmente atingidas pela pandemia, aqui demonstrado pela caracterização dos estados nordestinos. Aponta-se, portanto, para importância dos esforços dos governos para mitigar a suscetibilidade à COVID-19 com evolução para óbito, frente aos desafios e às condições assistenciais e socioeconômicas da região.

## Considerações finais

Há uma clara relação entre medidas sanitárias de distanciamento social e vacinação e redução de morbimortalidade da população idosa nos estados da Região Nordeste. Entretanto, as decisões dos governos estaduais da região não foram suficientes para mitigar o impacto da morbimortalidade entre as pessoas idosas em consequência da pandemia de COVID-19.

Há desafios anteriores à emergência sanitária quanto ao acesso e à qualidade da atenção especializada para essa população, com desigualdades entre os territórios, que foram ampliados na crise sanitária. Logo, reforça-se a necessidade de repensar políticas de saúde no sentido de um envelhecimento saudável.
